# Editorial: Antioxidant activities and induction production approaches (Physical, chemical and biotechnological) of plant-based bioactive nutrients

**DOI:** 10.3389/fnut.2026.1980184

**Published:** 2026-10-01

**Authors:** Jim Junhui Huang, Shaoling Lin

**Affiliations:** 1 Department of Food Science and Technology, Faculty of Science, National University of Singapore, Singapore, Singapore; 2 College of Food Science, Fujian Agriculture and Forestry University, Fuzhou, Fujian, China

**Keywords:** antioxidant activities, biotechnological approaches, chemical approaches, physical approaches, plant-based bioactive nutrients, production

## Introduction

Plant-based bioactive nutrients are naturally occurring compounds derived from microalgae, vegetables, fruits, and other photosynthetic organisms that provide health benefits beyond basic nutrition. Representative compounds include carotenoids, anthocyanins, polyphenols, flavonoids, and alkaloids, many of which possess potent antioxidant properties and may help prevent oxidative stress-related disorders, including cardiovascular/neurodegenerative diseases, and cancers. These compounds are increasingly used in food/nutraceutical industries to improve nutritional value, quality, and functionality. However, the enormous diversity of plants means that the metabolic profiles and biological activities of many species remain poorly characterized. Additionally, effective physical, chemical, and biotechnological strategies are needed to enhance biosynthesis, extraction, stability, bioavailability, and production of these valuable compounds. Therefore, this Research Topic aims to provide updated knowledge on the antioxidant activities and production approaches of plant-based bioactive nutrients.

## Metabolic profiles and bioactivities of plant extracts

This Research Topic investigated/reviewed the metabolic profiles and bioactivities of extracts from several plants that have received limited previous attention, including *Curcuma longa* (turmeric), *Hylocereus costaricensis* (red dragon fruit), *Nardostachys jatamansi*, black tea, *Matricaria chamomilla* (chamomile), and various culinary spices. Perez et al. investigated *Curcuma longa* containing abundant curcuminoids and phenolic compounds. Its extracts demonstrated antioxidant, neuroprotective, and anti-inflammatory activities, associated with acetylcholinesterase and lipoxygenase inhibition, respectively. Direito et al. found that *Hylocereus costaricensis* phenolic extracts contained high levels of phenolics and flavonoids, together with bioactive compounds like betanin, rutin, and ferulic acid. Zhang et al. studied the essential oil of *Nardostachys jatamansi* (NJEO). Hydrodistillation yielded 4.28% essential oil, where GC-MS identified 30 compounds, mainly terpenes and aromatic constituents. NJEO exhibited strong free-radical-scavenging activity, particularly against superoxide radicals, and effectively inhibited *Staphylococcus aureus* and *Escherichia coli*, with greater activity against Gram-positive bacteria. Molecular docking indicated that several constituents may interact with antioxidant-/ antibacterial-related protein targets, supporting the potential application of NJEO as a natural antioxidant/antimicrobial agent. Lu et al. compared Gougunao No. 2 (G2R) black tea with the traditional Gougunao group small-leaf cultivar (BDZ) using untargeted UPLC-HRMS metabolomics and antioxidant assays. G2R showed distinct metabolic profiles, enriching amino acid, flavonoid, and phenylpropanoid pathways. It contained higher levels of total phenolics and flavonoids and exhibited significantly greater ferric-reducing antioxidant power and hydroxyl radical-scavenging activity than BDZ, although ABTS and DPPH activities were not significantly different. These findings demonstrate that cultivar background strongly affects black tea composition and antioxidant properties and highlight metabolomics as a useful tool for quality evaluation and cultivar improvement. Ferhat et al. examined hydroethanolic extract of *Matricaria chamomilla* collected from semi-arid Algeria. LC-MS/MS identified several bioactive compounds, including caffeic acid, gallocatechin gallate, and apigenin-7-O-glucoside. Although the extract showed moderate antioxidant activity, it demonstrated strong antibacterial, antifungal, antidiabetic, anticholinesterase, and anti-inflammatory effects *in vitro* and *in vivo*. Molecular docking suggested interactions with breast cancer-related targets such as ERα and HER2. The extract also showed low acute toxicity in mice, suggesting potential applications in functional foods and therapeutic products. Ozler and Sanlier reviewed culinary spices as natural anti-aging agents for skin health. Spices like turmeric, ginger, rosemary, cinnamon, saffron, clove, thyme, black pepper, coriander, and cumin contain antioxidant and anti-inflammatory compounds that may reduce oxidative stress and inflammatory cytokines, inhibit matrix metalloproteinases, promote collagen synthesis, suppress melanin production, and protect against UV-induced damage. Nevertheless, most evidence comes from *in vitro*, animal, or topical studies, further well-designed human clinical trials are needed.

## Induction production approaches

This Research Topic also highlighted physical, chemical, and biotechnological approaches for improving the production, recovery, delivery, and functionality of plant-based bioactive nutrients.

## Physical approaches

Perez et al. developed a sequential green extraction strategy combining supercritical fluid extraction (SFE) and ultrasound-assisted extraction (UAE) to recover bioactive compounds from *Curcuma longa*. The process produced curcuminoid- and phenolic-enriched extracts with strong antioxidant, neuroprotective, and anti-inflammatory activities. Compared with conventional extraction, the combined approach significantly improved bioactive recovery and extraction efficiency. The extract was further formulated into a stable nanoemulsion that maintained its physicochemical properties for at least 15 days, demonstrating potential for functional foods, nutraceuticals, and therapeutic formulations.

## Chemical approaches

Direito et al. investigated phytosome encapsulation of *Hylocereus costaricensis* phenolic extract to improve biological activity and bioavailability. Although phytosomes encapsulated approximately 46% of phenolics, they retained antioxidant activity at a lower dose comparable to that of crude extract. In rat models, phenolic-enriched phytosomes markedly enhanced antihyperglycemic and anti-inflammatory effects, with efficacy comparable to metformin and stronger anti-inflammatory activity than the free extract. No detectable liver/kidney toxicity was observed, indicating that phytosomal delivery may be an effective strategy for improving the nutraceutical potential of plant-derived phenolics.

## Biotechnological approaches

Pei et al. developed selenium nanoparticles (DHP-SeNPs) stabilized with polysaccharide extracted from *Dendrobium huoshanense*. The optimized nanoparticles were stable and spherical, with improved thermal stability while retaining the structural characteristics of the polysaccharide. Compared with the original polysaccharide, DHP-SeNPs exhibited substantially stronger DPPH, hydroxyl radical, and ABTS^+^ scavenging activities and greater reducing power. In RAW264.7 macrophages, they showed no cytotoxicity, promoted cell proliferation, enhanced phagocytosis and scratch healing, and increased cytokine expression, including TNF-α, IL-1β, IL-6, and IL-10. These results demonstrate promising antioxidant and immunomodulatory potential for food and biomedical applications. Chi et al. developed a lotus-seed-based vegan yogurt using mixed lactic acid bacterial fermentation. The optimal ratio of *Lactobacillus bulgaricus, Lactiplantibacillus plantarum*, and *Streptococcus thermophilus* was 1:2:1, with fermentation at 38 °C for 7.5 h. The resulting product exhibited excellent water-holding capacity, texture, and sensory quality. Compared with dairy yogurt, it contained less fat and fewer calories but more dietary fiber. Fermentation generated a stable gel network while preserving important alkaloids, including neferine and nuciferine, suggesting potential functional benefits, particularly for sleep-related applications.

## Conclusions

Overall, this Research Topic demonstrates considerable potential of plant-based bioactive nutrients as sources of antioxidants and multifunctional health-promoting compounds. Combining advanced metabolic profiling with physical extraction, chemical encapsulation, and modern biotechnology can improve their production, stability, bioavailability, and biological effectiveness. These approaches provide valuable foundations for developing innovative functional foods, nutraceuticals, and biomedical products. Future research should focus on optimizing large-scale production, clarifying molecular mechanisms, improving bioavailability, establishing safety profiles, and conducting well-designed human clinical trials to translate these promising findings into practical health applications.

